# Supplementary material for: Crawling and Gliding: A Computational Model for Shape-Driven Cell Migration
Source: PLoS Comput Biol. 2015 Oct 21;11(10):e1004280. doi: 10.1371/journal.pcbi.1004280 (PMC4619082; doi:10.1371/journal.pcbi.1004280)
Supplement: S1 Code — (ZIP) [file pcbi.1004280.s012.zip › release/tst/doc/html/index.html]

Tissue Simulation Toolkit: Tissue Simulation Toolkit, v0.1.4.2


Tissue Simulation Toolkit
 0.1.4.1


- Main Page
- Namespaces
- Classes
- Files

Tissue Simulation Toolkit, v0.1.4.2

Welcome to the Tissue Simulation Toolkit, a library for two-dimensional simulations of Glazier and Graner's Cellular Potts model (Glazier and Graner, 1993).

The TST aims to provide a simple set of computational tools to get you started with Cellular Potts Simulations.

The current version of the TST includes example programs for the following published simulations:

- Differential adhesion driven cell sorting (Glazier and Graner, 1993)
- Cell elongation dependent vasculogenesis (blood vessel growth) (Merks et al., 2006)
- Contact-inhibition dependent vasculogenesis and angiogenesis (Merks and Glazier, 2005; Merks and Glazier, 2006; Merks et al, PLoS Comput Biol 2008)

The TST provides many recent extensions to the CPM, including

- Infinite number of PDE layers (forward Euler)
- Interaction of CPM cells and PDE (secretion, absorption)
- Chemotaxis
- Length and connectivity constraints

and visualization of:

- Cells, according to cell type or anything you wish
- Chemical fields, using color ramps and contour lines (level sets)

# Installation

The TST has been compiled on Windows XP, Windows 7 (minGW), MacOS X and Linux systems (GNU C++). The following libraries are required:

Qt (version 4) libpng and libz

For Unix, we also have an (obsolete) X11-only version, although compilation is easiest with the qmake tool shipped with Qt (see http://www.trolltech.com)

With Qt, simply type:

qmake make

If you want to use a different "main" program (examples are given: vessel.cpp and sorting.cpp), just change "TARGET" in the file "CellularPotts2.pro".

# Programs

A small Cellular Potts tutorial making use of the example programs sorting.cpp and vessel.cpp is provided by the document "exercises.pdf" included in the source package.

# Documentation

An automatically generated class documentation is included in html/index.html.

A tutorial on CPM programming will soon come out as: Josephine T. Daub and Roeland M. H. Merks. Cell-based computational modeling of vascular morphogenesis using Tissue Simulation Toolkit. In: Vascular Morphogenesis. Domenico Ribatti (Ed.) Methods in Molecular Biology, in press.

# References

- Glazier, J. A. and Graner, F. 1993. Simulation of the differential adhesion driven rearrangement of biological cells. Phys. Rev. E 47, 2128-2145.
- Merks, R. M. H., Brodsky, S. V., Goligorsky, M. S., Newman, S. A. and Glazier, J. A., 2006. Cell elongation is key to in silico replication of in vitro vasculogenesis and subsequent remodelling. Dev. Biol. 289, 44-54.
- Merks, R. M. H. and Glazier, J. A., 2005. A cell-centered approach to developmental biology. Phys. A. 352, 113-130.
- Merks, R. M. H. and Glazier, J. A., 2006. Dynamic mechanisms of blood vessel growth. Nonlinearity 19, C1-C10.
- Merks, R. M. H., Perryn, E. D., Shirinifard, A., and Glazier, J. A., 2008. Contact-inhibited chemotaxis in de novo and sprouting blood-vessel growth. PLoS Comput Biol 4(9): e1000163.


---

Generated on Thu Aug 14 2014 22:04:01 for Tissue Simulation Toolkit by  

 1.8.6
